# Assessment of surgical antimicrobial prophylaxis in Orthopaedics and Traumatology Surgical Unit of a Tertiary Care Teaching Hospital in Addis Ababa

**DOI:** 10.1186/s13104-017-2475-2

**Published:** 2017-04-20

**Authors:** Nitsuh Alemayehu Argaw, Kibruyisfawe Zewdie Shumbash, Alemseged Ayele Asfaw, Segewkal Hawaze

**Affiliations:** 1 Department of Pharmacology and Clinical Pharmacy, School of Pharmacy, College of Health Sciences, Addis Ababa University, P. O. Box 1176, Addis Ababa, Ethiopia; 2 Department of Surgery: Neurosurgery Unit, School of Medicine, College of Health Sciences, Addis Ababa University, P. O. Box 1176, Addis Ababa, Ethiopia; 3 School of Pharmacy, College of Public Health and Medical Sciences, Jimma University, P. O. Box: 378, Jimma, Ethiopia

**Keywords:** Preoperative antimicrobial prophylaxis, Postoperative antimicrobial prophylaxis, Surgical site infection, Tikur Anbesa Specialized Hospital, Ethiopia

## Abstract

**Background:**

Prophylactic antimicrobials have a starring role in prevention of surgical site infection. This study assesses the practice of surgical antimicrobial prophylaxis (SAP) and development of surgical site infection (SSI) based on patient chart review in patients who underwent surgery in the Orthopaedics and Traumatology Surgical Unit of Tikur Anbesa Specialized Hospital (TASH).

**Results:**

Majority of the patients 144 (72%) were males. 108 (54%) of the surgical wounds were clean and 63 (31%) were clean contaminated. 160 (80%) patients received preoperative prophylaxis, of these 153 (96%) received postoperative prophylaxis as well. 34 (17%) patients did not receive preoperative antimicrobial prophylaxis, while 6 (3%) patients had no record about preoperative antimicrobial prophylaxis. Among those who received preoperative antimicrobial prophylaxis the time of administration was not recorded in 87 (54%) of the patient charts and 36 (23%) patients had preoperative antimicrobial prophylaxis greater than 2 h prior to incision. Among the 188 (94%) patients that received postoperative antimicrobial prophylaxis; the duration of administration was more than 72 h in 114 (61%) patients, while only 8 (4%) received for less than 24 h after surgery. Ceftriaxone 309 (70%) was the most prescribed agent for prophylaxis. 32 (16%) patients developed surgical site of infection. Using odds ratio age equal to or above 50, clean contaminated and contaminated surgical wounds were not statistically associated with increased risk of SSI.

**Conclusion:**

Most patients who underwent surgery received prophylactic antimicrobials; nevertheless, the practice was not aligned with standard guidelines’ recommendations and patients developed surgical site infections.

## Background

SSI is a common postoperative problem and represents a substantial burden in terms of morbidity, mortality, quality of life, longer hospital stays, readmissions, and supplementary use of antimicrobials that can lead to antibiotic-resistant bacteria and increased cost of health services [1, 2]. There are many risk factors for SSI such as patient or operation characteristics [3].

Among the operation characteristics, SAP is crucial in preventing incidence of SSI [4]. SAP has become customary practice for contaminated and clean contaminated surgery [5]. Prophylactic antimicrobials are used when the risk of postoperative infection outweigh its risk; and antimicrobials are selected based on spectrum of activity, susceptibility of pathogens, duration of action, cost and other parameters [6].

Antimicrobials are valued 20–30% of hospital pharmacy budget [7] and nearly 30–50% of these antimicrobials are given for SAP [5]. Yet, 30–90% of these SAPs are inappropriate; most antimicrobials are either given at the wrong time, wrong dosage and wrong strength which results in increased antibiotic usage, increased costs, prolonged hospitalization, super infection, and antimicrobial resistance [5, 8, 9]. Moreover, SAP protocols should be revised, as cost of antimicrobials and resistance change [10].

Orthopaedic surgical procedures usually involve implants, which can facilitate infection due to contamination by exogenous sources (during the perioperative period) or thru eventual haematogenous spread of microorganisms (after perioperative period) [11, 12]. Furthermore, in orthopaedic surgical procedures infections are commonly detected within 2 years, following procedures; therefore, to classify as SSI, ‘the diagnosis must be made within 12 months of the procedure’ [13].

World Health Organization (WHO) report on burden of health care associated infection illustrates that the incidence rate of SSI in low and middle income countries ranges from 1.2–23.6% [14]. Different studies signpost inappropriate SAP as important cause for SSI [11, 15]. Moreover, studies on developing countries indicate that SAP is not delivered properly [16, 17]. In Ethiopia studies on practice of SAP are scant, but the existing studies on use of antimicrobials depict inappropriate practice and high encumbrance of infections [18–23]. The fact that no researches were conducted on the practice of SAP in the Orthopaedics and Traumatology Surgical Unit of TASH, high incidence of infections, antimicrobial resistance and associated costs indicated by previous studies dictated this research. Hence, this study was conducted with the objective of assessing SAP practice and rate of SSI in patients who underwent surgical procedures in the Orthopaedics and Traumatology Surgical Unit of TASH.

## Methods

### Study setting and study design

The study was conducted in the Orthopaedics and Traumatology Surgical Unit of TASH. TASH is the largest specialized and teaching hospital in Ethiopia. It has a total of 627 beds and gives service to 1000 patients per day. The surgical department consists of seven units: cardiothoracic and vascular surgery unit, general and endocrine surgical unit, neurosurgery unit, urology surgery unit, paediatrics surgical unit, Orthopaedics and Traumatology Surgical Unit, gynaecological surgical unit. The Orthopaedics and Traumatology Surgical Unit conducts teaching as well as surgeries, on average 806 major orthopaedic surgeries are done annually [24].

Institution based retrospective cross-sectional study was conducted to assess SAP practice and rate of SSI on patients who had surgical procedures in the Orthopaedics and Traumatology Surgical Unit of TASH from September 2012–September 2013. Data about prescribed medications and patient characteristics were collected from patient charts using a pre-tested data abstraction format. All adult patients that underwent orthopaedic surgery with no previous existing infection and antimicrobial treatment were included in the study. Furthermore clean, clean contaminated and contaminated types of wounds were included in the study while dirty wounds were excluded.

### Data collection and management

Data was collected by final year pharmacy students who were trained on how to collect SAP and SSI from patient charts using abstraction format in a uniform and comprehensive manner. The abstraction format contained parts for patient characteristics; types of surgical wound; antimicrobial agent; route and dose; administration time of preoperative prophylaxis; intraoperative (re-dose) prophylaxis; duration of postoperative surgical prophylaxis and development of SSI.

The assessment was done using prophylactic antibiotics in orthopaedic surgery [25] and clinical practice guideline for antimicrobial prophylaxis in surgery [6], the commonly adopted guidelines by the unit; to compare the findings with the standard recommendations. Completeness of data was checked; data was cleaned, coded, and entered in Epi Info^®^ version 3.5.1 for Windows (CDC, Atlanta, GA, USA). Then it was analysed using SPSS version 20.0 for Windows (SPSS Inc, Chicago, IL, USA). Descriptive statistics were generated and odds ratio (OR, 2 × 2 contingency tables) was performed.

### Operational definitions

#### Clean wound

An uninfected operative wound in which no inflammation is encountered and there is no entry into the respiratory, alimentary, genital, or urinary tract. Clean wounds are closed, if necessary are drained with closed drainage. Operative incisional wounds that follow non-penetrating trauma are included in this category if the above criteria are met [4].

#### Clean contaminated

Operative wounds in which respiratory, alimentary, genital, or urinary tracts are entered under controlled conditions and without unusual contamination. With no evidence of infection encountered or major break in technique [4].

#### Contaminated

Open, fresh accidental wounds. Operations with major breaks in sterile technique or gross spillage from the gastrointestinal tract; incisions in which acute, non-purulent inflammation has been encountered [4].

#### Surgical antimicrobial prophylaxis

A brief course of an antimicrobial agent administered to patient either before, during or after surgery with the aim of reducing microbial burden of intraoperative contamination to a level of overwhelm the host defiance [4].

#### Surgical site infection

An infection that occurs within a year of surgery and involves only the skin or subcutaneous tissue of the incision, and meets at least one of the following criteria: (1) purulent discharge from the superficial incision (2) superficial incision yields organisms from the culture or aseptically aspirated fluid, tissue, or from a swab, and pus cells are present (3) at least two of the following symptoms and signs: pain or tenderness, localized swelling, redness, heat and; (a) the superficial incision is deliberately opened by a surgeon to manage the infection, unless the incision is culture negative or (b) the clinician diagnoses a superficial incisional infection [26].

## Results

### Characteristics of patients

From the 200 patients who had surgical procedures, chart review indicated 144 (72%) patients as males. Mean age of patients was 33 ± 15 years and the age group 24–34 years represented the largest portion 74 (37%). 172 (86%) patients did not have any co-morbid condition, as shown on Table 1.

**Table 1:** Characteristics of patients (N = 200) that had surgery in the Orthopaedics and Traumatology Surgical Unit of TASH from September 2012–September 2013

Characteristics		N (%)
Sex	Male	144 (72)
	Female	56 (28)
Age in years	13–23	53 (27)
	24–34	74 (37)
	35–45	42 (21)
	46–56	11 (5)
	57–67	11 (5)
	>68	9 (5)
Co-morbid condition	Yes	28 (14)
	No	172 (86)

### Classification of surgical wound

We used, National Healthcare Safety Network, terminology for wound classification (see “Operational definitions” in Methods). Classification of the surgeries per types of surgical wounds revealed that majority of the surgical wounds were clean 108 (54%) followed by clean contaminated 63 (31%) and contaminated 29 (15%).

### Practice of surgical antimicrobial prophylaxis

Among the 200 patient charts analysed, 160 (80%) patients received preoperative antimicrobial prophylaxis among which 153 (96%) received postoperative antimicrobial prophylaxis as well. While 34 (17%) did not receive preoperative antimicrobial prophylaxis. There was no record about preoperative antimicrobial prophylaxis in 6 (3%) patient charts. There was no re-dose of antibiotics prophylaxis in 199 (99%) surgical procedures. Among the 200 patient charts analysed, 188 (94%) received postoperative antimicrobial prophylaxis. From the 34 (17%) patients who did not receive preoperative antimicrobial prophylaxis, 5 (15%) patients did not receive postoperative antimicrobial prophylaxis.

**Fig. 1:**
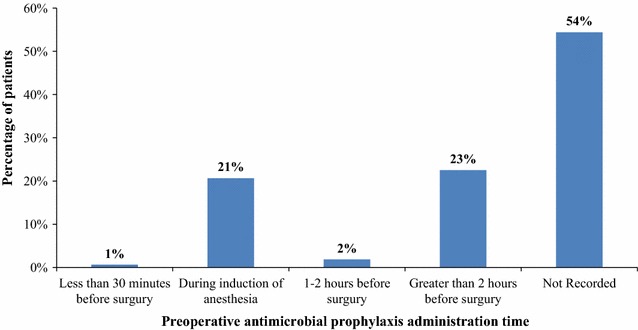
Time of preoperative antimicrobial prophylaxis (N = 160) in the Orthopaedics and Traumatology Surgical Unit of TASH from September 2012–September 2013

Among those who received preoperative antimicrobial prophylaxis the time of administration was not recorded in 87 (54%) of the patient charts. While 36 (23%) and 33 (21%) patients received preoperative antimicrobial prophylaxis greater than 2 h prior to incision and during induction of anaesthesia respectively as shown in Fig. 1. All prophylactic antimicrobial agents were administered via intravenous route.

**Fig. 2:**
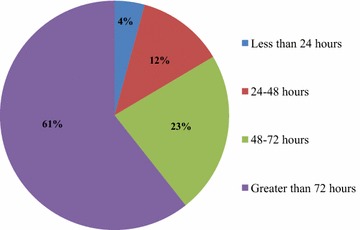
Duration of postoperative antimicrobial prophylaxis (N = 188) in the Orthopaedics and Traumatology Surgical Unit of TASH from September 2012–September 2013

From the 188 (94%) patients that received postoperative antimicrobial prophylaxis the duration of administration was more than 72 h after surgery in 114 (61%) patients, followed by 48–72 h in 43 (23%) patients. While only 8 (4%) patients received postoperative antimicrobial prophylaxis for less than 24 h after surgery, as indicated in Fig. 2.

### Antimicrobials prescribed as prophylactic agents

**Fig. 3:**
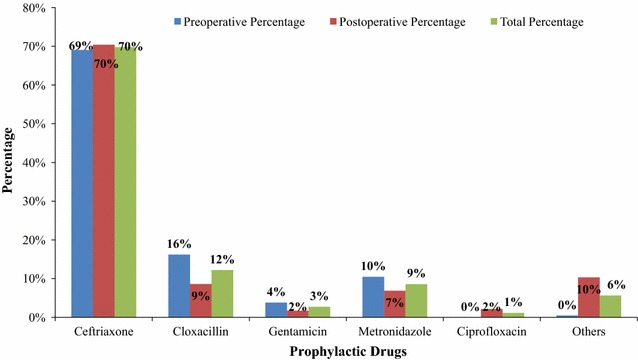
Antimicrobials prescribed as preoperative (N = 210) and postoperative (N = 233) prophylactic agents in the Orthopaedics and Traumatology Surgical Unit of TASH from September 2012–September 2013. *Others* ampicillin, vancomycin, amoxicillin–clavulanic acid and cephalexin

In this study 200 patients received a total of 443 antimicrobials as prophylactic agents. All antimicrobials were prescribed by orthopaedics and traumatology surgeons and residents. The most frequently prescribed antimicrobial agent was ceftriaxone 309 (70%) and the least prescribed was ciprofloxacin 5 (1%) both in the preoperative as well as in postoperative period as shown in Fig. 3. The prophylactic antimicrobial regimens included both single as well as combination regimens; single regimens in both preoperative and postoperative took the lion’s share. The most commonly prescribed regimen among the combination regimens was ceftriaxone plus metronidazole; and triple combinations were also used as shown in Table 2.

**Table 2:** Antimicrobial regimens prescribed in the Orthopaedics and Traumatology Surgical Unit of TASH from September 2012–September 2013

Antimicrobial regimen	Preoperative (N = 160) N (%)	Postoperative (N = 188) N (%)
Ceftriaxone	103 (64)	123 (65)
Cloxacillin	14 (9)	–
Ceftriaxone + gentamicin	8 (5)	–
Ceftriaxone + metronidazole	14 (9)	16 (9)
Ceftriaxone + cloxacillin	12 (7)	16 (9)
Ceftriaxone + ciprofloxacin	–	5 (3)
Ceftriaxone + metronidazole + cloxacillin	8 (5)	–
Ceftriaxone + gentamycin + cloxacillin	–	4 (2)
Others	1 (1)	24 (12)

*Others* ampicillin, vancomycin, amoxicillin–clavulanic acid and cephalexin

### Appropriateness of surgical antimicrobial prophylaxis

The Orthopaedics and Traumatology Surgical Unit of TASH does not have a dedicated guideline for administration of antimicrobial prophylaxis. Therefore, appropriateness of SAP practice was compared to internationally accepted guidelines: prophylactic antibiotics in orthopaedic surgery [25] and clinical practice guideline for antimicrobial prophylaxis in surgery [6], the commonly adopted guidelines by the unit.

Per these guidelines for clean orthopaedic procedures and those procedures not involving implantation of foreign materials, the need for antimicrobial prophylaxis is not well established. Despite this, out of the 108 patients who had a clean wound, 78 (72%) patients received preoperative antimicrobial prophylaxis. Moreover, clean contaminated and contaminated type of surgical wounds, need preoperative prophylaxis and therapeutic management is based on severity per the guidelines. Among 63 patients with clean contaminated wound who are eligible for preoperative antimicrobial prophylaxis, only 55 (87%) patients were on preoperative antimicrobial prophylaxis. Likewise, from the 29 patients that had contaminated wound only 27 (93%) received preoperative antimicrobial prophylaxis. Comparison of surgical antimicrobial prophylaxis with guidelines recommendations is given in Table 3.

**Table 3:** Comparison of surgical antimicrobial prophylaxis in procedures conducted in the Orthopaedics and Traumatology Surgical Unit of TASH from September 2012–September 2013 (N = 200) with guidelines recommendations

Prophylactic antibiotics	N (%)
Indicated and administered	82 (41)
Not indicated and not administered	30 (15)
Indicated but not administered	10 (5)
Not indicated but administered	78 (39)

Choice of antimicrobials was not consistent with the recommendations of the guidelines in all the patients that received prophylactic antimicrobials. Narrow spectrum antibiotics are indicated for clean contaminated and contaminated type surgical wound; despite this recommendation wide spectrum antimicrobials were used. The time of administration of preoperative antimicrobial prophylaxis was not recorded for 87 (54%) patients. Timing of preoperative antimicrobial prophylaxis was inappropriate in 39 (24%) of the patients. Besides, duration of postoperative antimicrobial prophylaxis was prolonged more than 24 h in 180 (96%) patients that received postoperative antimicrobial prophylaxis, against the recommendations of the guidelines.

### Surgical site infection developments

From the 200 patients include in this study 32 (16%) developed SSI within one year after surgery was done, of those 25 (78%) were males. There were 13 (41%) clean type of surgical wounds among the SSIs that developed, while the rest were clean contaminated and contaminated type of surgical wounds.

Of those patients that developed SSI 28 (87%) received preoperative antimicrobial prophylaxis. 22 (69%) patients who developed SSI were among those patients whose charts indicated not recorded time of preoperative prophylaxis and who were administered preoperative prophylaxis greater than 1 h before surgery. 31 (97%) patients that developed SSI had postoperative antimicrobial prophylaxis and 25 (78%) of the patients that had SSI received postoperative antimicrobial prophylaxis for more than 24 h. Odds ratio performed revealed that age equal to or above 50; and having clean contaminated and contaminated surgical wounds were not associated with increased risk of SSI, as shown in Table 4.

**Table 4:** Association of selected parameters with SSI in patients who underwent surgery in the Orthopaedics and Traumatology surgical unit of TASH from September 2012–September 2013

Variable	SSI	No SSI	OR	95% CI
Age				
<50	27	145	0.86	0.37–2.09
≥50	5	23		
Sex				
Male	25	119	1.47	0.64–3.03
Female	7	49		
Type of surgery				
Clean	13	95	0.53	0.31–1.12
Clean contaminated and contaminated	19	73		
Preoperative prophylaxis				
Yes	28	132	1.91	0.65–4.70
No and no record	4	36		
Preoperative prophylaxis administration time				
≤1 h	6	28	1.01	0.45–2.29
>1 h and not recorded	22	104		
Postoperative prophylaxis				
Yes	31	157	2.17	0.29–13.28
No	1	11		
Duration of postoperative prophylaxis				
≤24 h	2	6	2.07	0.51–6.31
>24 h	25	155		

## Discussion

Findings of this study showed that majority of the patients who underwent surgical procedures received antimicrobial prophylaxis before and/or after surgery. Similar level of SAP coverage observed was also reported in other studies [27, 28]. This signifies an attempt in the Orthopaedics and Traumatology Surgical Unit of TASH to prevent SSIs. In this study 29 (15%) patients received postoperative antimicrobial prophylaxis without a prior preoperative antimicrobial prophylaxis, the finding from a study in Tanzanian hospital was like the one noted in our setting [16]. Furthermore, SAP practice observed in our study was not parallel with recommendations of prophylactic antibiotics in orthopaedic surgery [25] and clinical practice guideline for antimicrobial prophylaxis in surgery [6]. Studies by Rehan et al. and Vessal et al. also depict such practices [10, 29], which might be the root cause for overusing antimicrobials for prophylaxis, besides the lack of evidence based protocol.

As it was the case with other studies [10, 29–31], ceftriaxone, a third-generation cephalosporin was the most frequently used antimicrobial agent in the Orthopaedics and Traumatology Surgical Unit. Contrary to the above finding, cefazolin and cefuroxime were used commonly in Qatar [32]. Guidelines recommend narrow spectrum antimicrobials, first or second generation cephalosporins (such as cefazolin and cefuroxime) or clindamycin and vancomycin, in case of β-lactam allergy and colonization with methicillin-resistant *Staphylococcus aureus* (MRSA) [3, 6, 25]. The practice of adhering to wide spectrum antimicrobials at TASH could be attributed to absence of first and second generation cephalosporins, low cost of ceftriaxone, absence of microbiologic data and lack of evidence based protocol for the setting.

Despite recommendation of different guidelines, time of preoperative prophylactic antimicrobial administration in the orthopaedics and traumatology surgical unit was not recorded in 87 (54%) patient charts. Documenting time of prophylactic antimicrobial administration is crucial to make sure that SAP was given in recommended period [6]. Poor documentation could be due to work overload on attending nurses, absence of separate sheet for recording time of administration and lack of awareness to record SAP administration time. Moreover, the recommended time of administration per prophylactic antibiotics in orthopaedic surgery [25] and clinical practice guideline for antimicrobial prophylaxis in surgery [6] is within 1 h prior to incision to achieve adequate protection. Despite this 36 (23%) patients received SAP more than 2 h before surgery, alike practices in other Sub Saharan counties and India [29, 33]. However, 55.8% of patients in Indonesia received SAP 30 min prior to incision [30].

Regarding postoperative antimicrobial prophylaxis, prophylactic antibiotics in orthopaedic surgery and clinical practice guideline for antimicrobial prophylaxis in surgery recommend, a single dose of antimicrobial agent for not more than 24 h [6, 25]. Other studies also pointed out that prolonged postoperative antimicrobial prophylaxis doesn’t provide any supplementary benefit and indicated that drug resistant pathogens were noted in prolonged prophylaxis [2, 31]. In the face of this, 180 (96%) patients received postoperative antimicrobial prophylaxis for more than 24 h after surgery in the Orthopaedics and Traumatology Surgical Unit, akin to numerous studies which reported prolonged postoperative antimicrobial administration [1, 8, 10, 31, 32] and study in Gonder University Hospital where duration of surgical prophylaxis was 3–5 days [20].

The incidence of SSI in our study was lower compared to studies in Tanzania (19%) [16] and Nigeria (23.6%) [17]; but it was higher compared to Egypt (8.26%) [34] and India (14%) [29]. Comparing the incidence of SSI in the Orthopaedics and Traumatology Surgical Unit with other studies within the country, incidence was higher than the finding from a study conducted in TASH by Taye (14.8%) [22]; while incidence was lower compared to a study in Gondar University Specialized Hospital with SSI incidence of 21% [18]. The variation in the incidence of SSI in this study in contrast with other studies could be, due to antimicrobial agent choice, susceptibility pattern of pathogens, operation theatre setup and difference in study design and time.

Additionally, a study by Amoran et al. put forward other reasons such as use of out dated equipment, limited ventilation in the operating room or limited application of infection control measurement for development of SSIs [35], which could be the case in our study setting besides the improper SAP practice observed. These reasons proposed by Amoran et al. could also explain the discrepancy in the association between SAP (preoperative antimicrobial prophylaxis and postoperative antimicrobial prophylaxis) and decreased incidence of SSI.

## Conclusion

As a synopsis, this study indicated that majority of patient who underwent surgical procedures in the Orthopaedics and Traumatology Surgical Unit received SAP. Nevertheless, the practice was not aligned with standard guidelines. Wrong antimicrobial choice; inappropriate timing of administration of preoperative antimicrobial prophylaxis and prolonged duration of postoperative antimicrobial prophylaxis; and lack of proper documentation pertaining to patient charts and patient progressive notes were problem identified in the SAP practice.

This study indicated the need for evidence based guideline for the practice of SAP in the unit taking resistance pattern into consideration. Isolation of common microorganism responsible for SSI, adherence to narrow spectrum antimicrobials, preparation of separate sheet for recording SAP, responsibility to complete patient charts and progressive notes, and efforts from the hospital pharmacy to ensure availability and affordability of drugs of choice are encouraged.

## Limitations

Certain limitations were encountered while conducting this study. One of the limitations was unavailability of patient charts at time of data collection and exclusion of patient charts for those who are younger than 13. Thus, these factors might have affected the overall picture of this study on SAP in the Orthopaedics and Traumatology Surgical Unit of TASH. Other limitations encountered were loss of emergency orthopaedics and traumatology surgery logbook which could have had an important value (to compare with the elective surgeries), lack of proper follow-ups by patients, lack of progressive notes by attending physician and lack of microbiological screening of SSIs.

## Abbreviations

CI: confidence interval
MRSA: methicillin-resistant *Staphylococcus aureus*
SAP: surgical antimicrobial prophylaxis
SSI: surgical site infection
TASH: Tikur Anbesa Specialized Hospital
RR: relative risk

## References

[1] Gandage MG, Reddy PN, Shirsand SB, Ali SH, Kalyani B, Jeevangi VM (2013). Assessment of antibiotics prescription in surgical prophylaxis in a teaching hospital. RGUHS J Pharm Sci.

[2] Gagliardi AR, Fenech D, Eskicioglu C, Nathens AB, McLeod R (2009). Factors influencing antibiotic prophylaxis for surgical site infection prevention in general surgery: a review of the literature. Can J Surg.

[3] Scottish Intercollegiate Guidelines Network (SIGN). SIGN Guidelines 104. Antibiotic prophylaxis in surgery. A national clinical guideline. Edinburgh: SIGN; 2008. http://www.sign.ac.uk/pdf/sign104.pdf. Accessed 2 Apr 2013.

[4] Mangram AJ, Horan TC, Pearson ML, Silver LC, Jarvis WR (1999). Guideline for prevention of surgical site infection, Hospital infection control practices advisory committee. Infect Control Hosp Epidemiol.

[5] Munckhof W. Antibiotics for surgical prophylaxis. Aust Prescr. 2005;28:38–40. http://www.australianprescriber.com/magazine/28/2/38/40. Accessed 19 Apr 2014.

[6] Bratzler DW, Dellinger EP, Olsen KM, Perl TM, Auwaerter PG, Bolon MK (2013). Clinical practice guidelines for antimicrobial prophylaxis in surgery. Am J Health Syst Pharm.

[7] Gaynes R (2007). Antimicrobial Use in hospitals: managing a medical treasure. Med Gen Med.

[8] Mistry V, Pandya A, Chaudhari J, Sondarva D, Pillai A, Hotchandani S (2013). Use of antimicrobial prophylaxis in clean elective orthopedic surgical procedures and identifying common infective organisms. Int J Med Sci Public Health.

[9] Queiroz R, Grinbaum RS, Galvão LL, Tavares FG, Bergsten-Mendes G (2005). Antibiotic prophylaxis in orthopedic surgeries: the results of an implemented protocol. Braz J Infect Dis.

[10] Vessal G, Namazi S, Davarpanah MA, Foroughinia F (2011). Evaluation of prophylactic antibiotic administration at the surgical ward of a major referral hospital, Islamic Republic of Iran. EMHJ.

[11] van Kasteren ME, Manniën J, Ott A, Kullberg BJ, de Boer AS, Gyssens IC (2007). Antibiotic prophylaxis and the risk of surgical site infections following total hip arthroplasty: timely administration is the most important factor. Clin Infect Dis.

[12] Periti P, Mini E, Mosconi G (1998). Antimicrobial prophylaxis in orthopaedic surgery: the role of teicoplanin. J Antimicrob Chemother.

[13] Greene LR, Mills R, Moss R, Sposato K, Vignari M. An APIC guide. Guide to the elimination of orthopaedic surgical site infections. Washington, DC: APIC; 2010. http://www.apic.org/Resource_/EliminationGuideForm/34e03612-d1e6-4214-a76b-e532c6fc3898/File/APIC-Ortho-Guide.pdf. Accessed 22 Apr 2013.

[14] World Health Organization (WHO). Report on the burden of endemic health cared-associated infection worldwide: a systematic review of the literature. Geneva: WHO; 2011. http://whqlibdoc.who.int/publications/2011/9789241501507eng.pdf. Accessed 22 Apr 2014.

[15] Steinberg JP, Braun BI, Hellinger WC, Kusek L, Bozikis MR, Bush AJ (2009). Timing of antimicrobial prophylaxis and the risk of surgical site infections: results from the trial to reduce antimicrobial prophylaxis errors. Ann Surg.

[16] Eriksen HM, Chugulu S, Kondo S, Lingaas E (2003). Surgical-site infections at Kilimanjaro Christian Medical Center. J Hosp Infect.

[17] Ameh EA, Mshelbwala PM, Nasir AA, Lukong CS, Jabo BA, Anumah MA (2009). Surgical site infection in children: prospective analysis of the burden and risk factors in a sub-Saharan African setting. Surg Infect (Larchmt).

[18] Kotisso B, Aseffa A (1998). Surgical wound infection in a teaching hospital in Ethiopia. East Afr Med J.

[19] Amenu D, Belachew T, Araya F (2011). Surgical site infection rate and risk factors among obstetric cases of jimma university specialized hospital, southwest ethiopia. Ethiop J Health Sci.

[20] Abula T, Kedir M (2004). The pattern of antibiotic usage in surgical in-patients of a teaching hospital, Northwest Ethiopia. Ethiop J Health Dev.

[21] Messele G, Woldemedhin Y, Demissie M, Mamo K, Geyid A (2009). Common causes of nosocomial infections and their susceptibility patterns in two hospitals in Addis Ababa. Ethiop J Health Biomed Sci.

[22] Taye M (2005). Wound infection in Tikur Anbessa hospital, surgical department. Ethiop Med J.

[23] Mengesha RE, Kasa BG, Saravanan M, Berhe DF, Wasihun AG (2014). Aerobic bacteria in post surgical wound infections and pattern of their antimicrobial susceptibility in Ayder Teaching and Referral Hospital, Mekelle, Ethiopia. BMC Res Notes.

[24] Wamisho BL, Zewde W (2006). Major orthopaedic procedures: 17 Year Trends. East Cent Afr J Surg.

[25] Prokuski L (2008). Prophylactic antibiotics in orthopaedic surgery. J Am Acad Orthop Surg.

[26] Burton F. Preventing surgical site infections. Wound essentials. 2007;2:124–31. http://www.wounds-uk.com/wound-essentials/wound-essentials-2-preventing-surgical-site-infections. Accessed 25 Apr 2014.

[27] Bailly P, Lallemand S, Thouverez M, Talon D (2001). Multicentre study on the appropriateness of surgical antibiotic prophylaxis. J Hosp Infect.

[28] Bull AL, Russo PL, Friedman ND, Bennett NJ, Boardman CJ, Richards MJ (2006). Compliance with surgical antibiotic prophylaxis-reporting from a statewide surveillance programme in Victoria, Australia. J Hosp Infect..

[29] Rehan HS, Kakkar AK, Goel S (2010). Pattern of surgical antibiotic prophylaxis in a tertiary care teaching hospital in India. Int J Infect Control.

[30] Radji M, Aini F, Fauziyah S (2014). Evaluation of antibiotic prophylaxis administration at the orthopedic surgery clinic of tertiary hospital in Jakarta, Indonesia. Asian Pac J Trop Dis.

[31] Kigera JWM, Gakuo LN (2013). Is there a role for prolonged post-operative antibiotic use in primary total hip arthroplasty in the African setting?. SA Orthop J.

[32] Abdel-Aziz A, El-Menyar A, Al-Thani H, Zarour A, Parchani A, Asim M, et al. Adherence of surgeons to antimicrobial prophylaxis guidelines in a tertiary general hospital in a rapidly developing country. Adv Pharmacol Sci. 2013;2013:842593. http://www.hindawi.com/journals/aps/2013/842593. Accessed 14 Apr 2014. 10.1155/2013/842593 PMC3885161 24454349

[33] Fehr J, Hatz C, Soka I, Kibatala P, Urassa H, Battegay M (2006). Antimicrobial prophylaxis to prevent surgical site infections in a rural sub-Saharan hospital. Clin Microbiol Infect.

[34] Afifi IK, Baghagho EA (2010). Three month study of orthopaedic surgical site infections in an Egyptian University hospital. Int J Infect Control..

[35] Amoran OE, Sogebi AO, Fatugase OM (2013). Rates and risk factors associated with surgical site infections in a tertiary care center in South-Western Nigeria. Int J Trop Dis Health.

